# From combustion to consequence: respiratory health concerns from primary and aged smoke at the wildland–urban interface

**DOI:** 10.3389/fpubh.2026.1763671

**Published:** 2026-03-16

**Authors:** Veronica L. Penuelas, David D. Lo

**Affiliations:** 1 Division of Biomedical Sciences, School of Medicine, University of California, Riverside, Riverside, CA, United States; 2 BREATHE Laboratory, Riverside, CA, United States

**Keywords:** aged smoke, PAHs, primary organic aerosols, respiratory health, secondary organic aerosols, smoke exposure, wildland fires, wildland-urban interface

## Abstract

As climate conditions worsen, wildfires are occurring more often, scorching millions of acres of land and carrying both flames and smoke into urban environments. The resulting smoke emissions from fires in the wildlands, as well from fires crossing into urban spaces, are highly toxic with a chemical composition and concentration changing from fire to fire, depending on material burnt and oxygen availability. Furthermore, as smoke ages and travels, it undergoes a process of oxidation where it converts from primary to secondary organic aerosols. Unfortunately, due to the complex nature of wildfire smoke, studying the health effects from smoke exposure has proven difficult. Currently, literature highlights the developments of various cardiovascular, respiratory, neurological, and mental health conditions from chronic or acute exposure, but the disrupted biological pathways, biomarker expression, and resulting pathophysiology of smoke toxin exposure related disease development remain unknown. In this review, we discuss commonly reported adverse health outcomes from wildland and wildland urban interface smoke exposures with a primary focus on respiratory conditions. Furthermore, we address the changing composition of primary versus secondary, or aged, smoke and how it relates to the wildfire public health concern. Lastly, we aim to delineate the actions needed to correlate chronic disease development to smoke toxin exposure and highlight the need for a reliable experimental system to study these unanswered questions.

## 1 Background

### 1.1 Introduction to wildland fires and wildland-urban interfaces

Climate conditions are worsening globally, leading to much higher temperatures in some areas and longer winters in others (1). In the Western United States, temperatures have reached an all-time high, with longer lasting fire seasons (2). Alongside increasing temperatures, population density has grown significantly, leading to an increased demand for resource availability and the need for more infrastructure and expanded housing developments across the nation. Unfortunately, in correlation to warmer weather, lasting droughts, and heightened fire seasons, we are experiencing a greater prevalence of wildfires each year (1–3). Because of this, firefighters and local civilians are chronically and repeatedly exposed to fires for long periods of time, breathing in the toxic contents of the wildfire smoke emissions (4–6).

In the context of public health, it is important to distinguish between the various wildfire types – namely, wildland fires (WLF) and wildland-urban interfaces (WUI). In WLF, organic material is burnt, ranging from plant life to animal life. On the other hand, the WUI includes both organic materials and various housing and construction materials – such as wood, plastics, metals, cleaning supplies, gasoline, chemicals, fiberglass, and many other contents found in homes and work sites (7, 8). The materials burnt in WUI fires are similar to the components found in overseas military burn pits, which contain the aforementioned items in addition to medical waste, human waste, and ammunition (9). It is understood that veterans exposed to these burn pits are met with an increased risk of developing adverse respiratory symptoms and consequently, the development of these symptoms may be associated with downstream long-term progressive disease development, such as chronic obstructive pulmonary disease (COPD), interstitial lung disease (ILD), constrictive bronchiolitis, asthma, and fibrosis in exposed veterans (9–11). These associations further raise concern regarding the state of exposure to different fire-type emissions, highlighting the dangers of exposure to the toxins that arise from such complex burns. However, because of the many materials found in differing fire types, the composition of smoke emissions is extremely complex, making it difficult to pinpoint specific pathologies resulting from smoke exposure (6, 9, 12–15). Consequently, this may result in various underlying toxic effects in each exposed person, especially as smoke ages and travels (16–18).

During the 2024 and 2025 wildfire seasons, fires in Canada and the western United States made national news, threatening land and structures across millions of acres (Table 1). For about a six-month time frame, fires across Southern California decimated just under 200,000 acres of land. These included the Palisades, Eaton, Line, Bridge, and Airport fires in Los Angeles, Orange, Riverside, and San Bernardino Counties. Unfortunately, they wiped out land across many national forests, including the Cleveland and Angeles National Forests, and destroyed over 36,000 structures (19). Following this, Canadian wildfires burned 21.7 million acres of land from May through September during the 2025 season (20), displacing both human and animal populations. Furthermore, fires in Arizona decimated native land and land surrounding the Grand Canyon. Groves of White Sage plants were the primary target of one of these burns, earning the name of White Sage Fire. In Native American folklore, burning sage is meant to cleanse spaces of negativity and bad spirits (21), leading many Indigenous people to believe that this fire was a warning from Mother Nature, shedding light on human-made disturbances in nature and cleansing herself from it. Unfortunately, as native plants are burned down in wildfires, this habitat loss is often replaced with non-native or invasive species (21, 22), which can lead to higher prevalences of wildfires, such as the addition of eucalyptus in California – an easily ignitable plant species (23). Despite the fires’ origins or folkloric backgrounds, they can each be categorized as either WLF or WUI, as shown in Table 1. Concomitantly, each fire shares the common theme of direct or indirect smoke exposure to surrounding populations for weeks to months at a time, resulting in the development of various health problems from continuous and chronic exposure to the toxins within the smoke.

**Table 1:** Wildfire details from the 2024–2025 season.

2024–2025 wildfire season in parts of North America			
Wildfire	Duration	Acres Burned	WLF/WUI
Palisades Fire, LA County, CA, United States	31 days	23,448	WUI
Eaton Fire, LA County, CA, United States	25 days	14,021	WUI
Line Fire, San Bernardino County, CA	110 days	43,978	WLF
Bridge Fire, LA and San Bernardino Counties, CA	79 days	56,030	WLF
Airport Fire, Orange and Riverside Counties, CA	27 days	23,526	WUI
White Sage Fire, Coconino County, AZ	68 days	58,985	WLF
Canadian Fires, Across most Canadian Provinces*	Ongoing as of 12/5/2025	21,700,000	Both

*Canadian provinces include British Colombia, Alberta, Ontario, Manitoba, Saskatchewan, Yukon, Nova Scotia, Newfoundland/Labrador, Quebec, and New Brunswick (71).

### 1.2 Adverse health effects, aged smoke, and toxic particulates in wildfire emissions

Commonly reported symptoms from wildfire smoke exposures include respiratory, cardiovascular, mental health, and other neurological symptoms (4, 5, 24, 25). In terms of adverse respiratory symptoms, individuals acutely exposed to fires often experience coughing, wheezing, and phlegm development (25), with some exhibiting more critical complications, such as dyspnea and decreased lung function (26), indicating either an inflammatory response or physiological changes from airway remodeling or within the lung parenchyma. Reports show that hospitalization increases during fire season largely due to adverse symptoms, such as asthma development, stroke, and heart attacks (26, 27). The underlying mechanisms of disease triggered by WLF and WUI exposure remain widely unknown; however, harmful gaseous, aromatic toxins and particulates released within the smoke can trigger a cascade of pathophysiological developments.

Aromatics, such as polycyclic aromatic hydrocarbons (PAHs), are biologically active chemicals (28) found in coal, fossil fuels, and crude oil. They react with high temperatures and release into the atmosphere (29), making them one of the most common emissions found in fire smoke. PAH’s present in smoke in either particulate or gaseous phases, or as non-volatile or volatile (30), such as volatile organic carbons (VOCs). They are considered highly toxic, with PAHs such as benzene, toluene, naphthalene, fluorene, retene, phenanthrene, and anthracene – found in smoke – considered carcinogenic, immunogenic, or toxicogenic (30–32). As material burns, some PAHs are released as particulate matter (PM) into the atmosphere in fine (<2.5 μm) or ultrafine (<0.1 μm) particles (PM_2.5_ and PM_0.1_ respectively). Their smaller size make the particulates easier to inhale and consequently, ultrafine particulates can bypass the use of some respiratory protective equipment and embed into the lungs, increasing the risk of pulmonary toxicity (4, 12, 13, 31, 33–35). PMs found in WUI smoke exposure consist broadly of black, elemental, and organic carbons (36, 37); however, the specific carbonaceous composition of PMs from wildfires varies depending on their size, the fire’s fuel type, and oxygen availability, leading to complex profiles, and increasing the toxicity of their exposure (38). Furthermore, these toxins remain within the wildfire smoke in the atmosphere for long periods of time, swept up by wind currents that spread smoke over thousands of miles.

**Figure 1:**
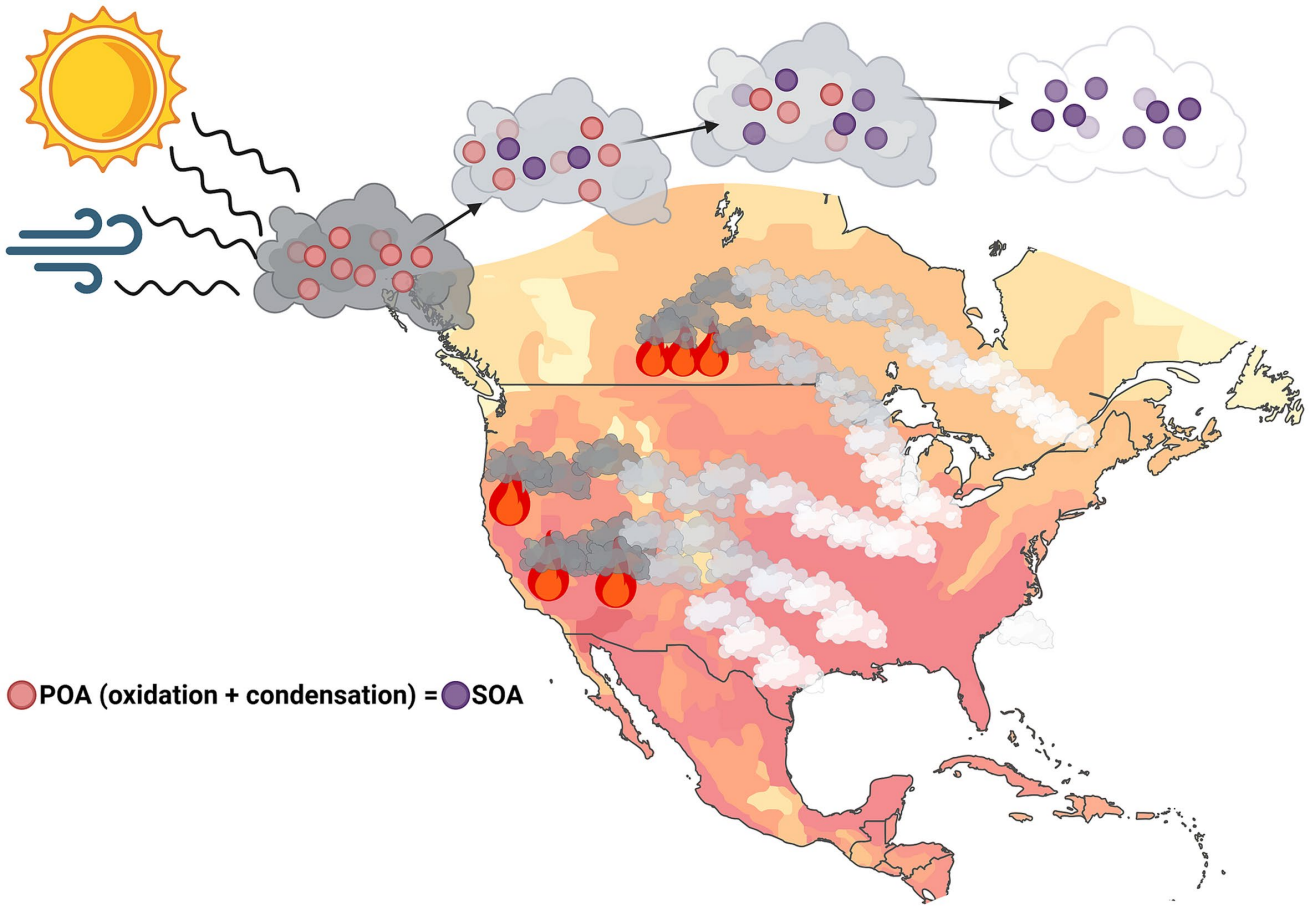
Primary to aged-smoke reaction. Primary organic aerosols released by wildfire smoke undergo a chemical reaction when exposed to sunlight and atmospheric gases, resulting in secondary organic aerosols (SOA). These SOAs can travel in smoke for days to weeks at a time in unknown concentrations. Created in BioRender, https://BioRender.com/0iaty4d (Penuelas, 2026).

As smoke travels, it goes through a process of oxidation and condensation from exposure to atmospheric gases and sunlight. This results in a change from primary organic aerosol (POA) to secondary organic aerosol (SOA) (Figure 1), with over 97% of fine particulates under 1 μm identified as SOA, or aged smoke, in smoke clouds that have persisted in the atmosphere for multiple days (39, 40). Health effects specifically resulting from SOA are not yet fully understood; however thorough analysis of the constituents that make up these organic aerosols (OA), such as PAHs and therefore, PMs, provide insight into adverse respiratory and other health detriments from SOA exposure. Furthermore, particulates in the ambient air and from other toxic environmental exposures may embed into the lungs of those exposed, potentially resulting in long-term and irreversible pulmonary damage (41–44). This correlation further highlights the concerns and need for understanding of adverse health outcomes directly from SOA/aged smoke exposure.

### 1.3 WLF and WUI: a public health concern

Understanding the adverse effects of exposure to particulate and gaseous matter from WUI smoke is imperative as these toxins are active contributors to inflammation (28, 45) which lead to long-term smoke-toxin-exposure-related-disease-development (STERDD). However, due to varying adverse health outcomes from exposure, a working and well-controlled *in vivo* experimental system that examines the direct effects of WUI and WLF exposure has not yet been developed. This forces researchers to rely on health reports generated from exposures to these toxins outside of wildfires, leading to inexact conclusions of how wildfires affect the population’s health. Unfortunately, this limits our understanding of what is happening within the body. Both acute and chronic exposure could disrupt normal biological and homeostatic mechanisms within the body – the detriments of which are driving disease development and physiological changes in organ systems. This has become a major public health concern, especially as a majority of metropolitan areas in the United States see an increased population every year (46). While proximity to wildfire hotspots is predictably a major environmental hazard, transport of toxic combustion products on the winds across thousands of miles also exposes far greater populations to these effects. In this review, we aim to (a) delineate precisely where the gaps in our knowledge on wildfires and the resulting health consequences are and (b) address how to improve upon scientific advancements to further understand the underlying physiological consequences as a direct result of fire smoke exposure.

## 2 Literature review methods

### 2.1 Keywords and search results

Literature reviews were conducted via PubMed and Google Scholar between October 3rd, 2025, through February 11th, 2026, for relevant articles. Keywords used included “wildland urban interface,” “wildfire smoke exposure,” “smoke emissions,” “respiratory health outcomes from smoke exposure,” “chronic health outcomes from smoke exposure,” “secondary organic aerosolized smoke,” “health effects from SOA,” “particulate matter in smoke,” “PAHs from smoke,” and “volatile organic carbons,” with search results provided in Table 2. Due to the high number of returned results, we prioritized papers that provided a public health outlook on wildfire and toxic smoke component exposures. In this paper, we include peer-reviewed data from original research, case studies, and review articles and reports. Despite the existence of many wildfire-health-effect-related articles, very little was found regarding original experimentation on the underlying biological effects of exposure – a major concern highlighted throughout this review – and therefore we relied heavily on other wildfire review articles to address these knowledge gaps.

**Table 2:** Returned results from source search by keyword.

Literature review search results		
Keywords	PubMed	Google Scholar
Wildland urban interface	*N* = 195	*N* = 39,500
Wildfire smoke exposure	*N* = 717	*N* = 48,900
Smoke emissions	*N* = 5,517	*N* = 807,000
Respiratory health outcomes from smoke exposure	*N* = 1,319	*N* = 535,000
CHRONIC health outcomes from smoke exposure	*N* = 1,310	*N* = 798,000
Secondary organic aerosolized smoke	*N* = 138	*N* = 7,140
Health effects from SOA	*N* = 494	*N* = 107,000
Particulate matter in smoke	*N* = 32,248	*N* = 454,000
PAHs from smoke	*N* = 5,727	*N* = 95,000
Volatile organic carbons	*N* = 6,140	*N* = 667,000

### 2.2 Inclusion and exclusion criteria

Papers that were excluded from this review were those focused primarily on cigarette smoke, tobacco, marijuana, vaping, and the use of other e-cigarettes. Papers included directly addressed wildfire smoke, wildland urban-interface smoke, SOA versus POA, particulate matter and hazardous air pollutants in smoke emissions and their overall effects on human health outcomes. Furthermore, we included studies and reports on other environmental exposure types [i.e., such as diesel emission exposure and other occupational exposures (42, 47–49)] which provided information regarding unsafe chemical exposure from fires and original research on underlying biological effects triggered by the exposures. The purpose of this inclusion is to address the lack of similar studies in wildfire research. Lastly, many sources returned in the keyword search were either not relevant for our review paper, were not peer-reviewed articles, were abstracts, or were duplicates of articles we already found and therefore were excluded from this review.

## 3 Discussion of wildfire research concerns

### 3.1 Population density and epidemiology reports in wildfire research

The United States (U.S.) is seeing a significant population increase in major metros from each yearly census. Indeed, from 2023 to 2024, 88% of the United States’ 387 urban-metro cities saw an increased population density that outpaced the average rate of population growth across the entire U.S. (46). As we see these growth patterns, the demands for infrastructure and more housing developments increase, leading to a higher demand for and over-exploitation of natural resources (21). Concomitantly, warmer climates across the United States increase the risk of fires and leads to heightened fire seasons. With this, we are witnessing more wildfires crossing into the urban interface, exposing the surrounding populations and traveling over hundreds of miles, affecting populations both within and outside of wildfire locations. Furthermore, hospitals are experiencing an increase in admissions during fire seasons with patients exhibiting neurological, respiratory, and cardiovascular related symptoms (4, 5, 24, 25).

Literature within the field of wildfire smoke exposure relies heavily on population epidemiology reports, where increased prevalence of acute upper respiratory symptoms from WLF exposure are noted (4–6, 12–15, 25). Upon repeated smoke exposures, many personnel, mainly firefighters, face increased risk of developing prolonged respiratory diseases, such as asthma, bronchoconstrictive conditions, COPD, peribronchiolar fibrosis, and lung cancer (4, 12, 13). Furthermore, fire retardant substances contain perfluoroalkyl and polyfluoroalkyl substances (PFAS), otherwise known as forever chemicals. As firefighters are repeatedly exposed to fires and fire retardants, they are directly and repeatedly exposed to higher concentrations of PFAS, which has been linked to the development of adverse health symptoms (49, 50). Indeed, Beaucham et al. (49) reported increased levels of PFAS in blood serum from firefighters who battled the 2023 Maui, Hawaii fires and consequently, many firefighting personnel were advised to seek additional medical screening due to the unsafe levels of measured PFAS. These studies are important as they address the concerns associated with repeated fire exposures for firefighters and other outdoor occupational workers who are exposed more consistently to fire smoke during active fire seasons (48, 49). However, measuring markers in blood serum obtained from these individuals provides unclear results as other exposure types or comorbidities that may impact these levels cannot be ruled out. Furthermore, the negative health outcomes vary greatly from person to person, a problem that is largely attributed to the complexity of smoke composition and conditions. As fire conditions and fuel/oxygen availability vary, the concentration of toxins from fire emissions changes (17, 51), indicating that specific toxins result in specific diseases. Likewise, as this variation in negative health outcomes becomes more prevalent with smoke toxins triggering inflammation, normal biological processes within the body become disrupted, paving way for specific STERDD, including progressive long-term lung pathology. However, which biological processes in the body are disrupted from wildfire smoke exposures remain undiscovered.

As population density increases and natural resources are wiped out or replaced with invasive species, the risk of wildfire occurrence becomes greater. With more fires and growing communities, hospitals become heavily burdened with smoke exposure victims throughout the duration of the fire season, depleting healthcare resources and increasing the demand for more medical facilities and professionals. Higher average hospital admissions coincides with increased smoke exposure related deaths, which averages between 40,000–71,000 yearly deaths from wildfire smoke exposure in North America (52). Increasing death tolls further highlight the importance for researchers and medical professionals alike to understand the adverse effects caused by these harmful emissions and better treat affected patients. Unfortunately, because of WUI fire complexity, studying the health outcomes from various smoke exposure types requires an experimental set-up of an equally complex nature. However, the demands for such an experimental set-up continue to increase as new pathophysiological mechanisms become prevalent in the context of WLF and WUI smoke exposure, with focus on correlations between specific toxins and disease outcomes.

### 3.2 Wildfire smoke toxins and their biological consequences

Air sampling of downwind smoke reveals excessive presence of nitrous acid (HONO), a major pollutant found in SOA/aged smoke that is the result of photolytic nitrate and nitrogen dioxide (NO_2_) uptake (40). Through direct environmental measuring and in-lab testing, HONO has been found to have mutagenic properties, leading to DNA damage (53, 54). Furthermore, preclinical animal models exposed to HONO demonstrate detrimental changes in lung function and the development of asthma-like symptoms from long-term exposure (55). These findings are consistent with human-based research, where HONO measurements (HONO produced by indirect combustion from gas stoves/ovens indoors) were conducted in the homes of consenting participants and compared alongside their lung function (56). Furthermore, as smoke is emitted from fires, its nitrite components oxidize rapidly (approximately <4 h), and the gaseous to particulate to organic ratio of its oxidized components are fairly matched (57), however, likely differ from fire to fire. Under long-term aging conditions, the organic aerosol ratio increases, and significantly more SOA is seen from fire-type smoke emissions (58). Considering the known health effects from SOA and its HONO constituents, it is likely that inhalation of aged wildfire smoke can result in the development of major adverse, and potentially irreversible, respiratory conditions.

OA makes up a large portion of environmental pollutants. In fact, research conducted from environmental sampling indicates that OA makes up more than half of atmospheric pollutants when compared to its inorganic counterpart. Additionally, approximately 58% of all OA are SOA (51). The higher percentage of SOA over POA indicates that as these toxic organic byproducts are released into the atmosphere, they oxidize quickly. Indeed, as POAs are released, they form bonds with hydroxyl radicals which incite oxidative stress and cause adverse health outcomes, leading to a ratio of SOAs that changes seasonally (40, 51, 59). Oxidative stress is known to cause adverse effects within the body, such as damaging DNA and proteins. The presence of oxidative stress can trigger inflammatory conditions and other diseases, such as the development of cancer, neurological concerns, and cardiovascular conditions such as hypertension and atherosclerosis (60). Therefore, exposure to SOA from wildfire smoke, both at high-dose from close exposure and low-dose from dispersed smoke, leads to a range of pathophysiological developments that are likely partially triggered by oxidative stress from bound hydroxyl radicals.

In an atmospheric exposure sampling study performed by O’Dell et al., over 32 hazardous air pollutants (HAPs) were identified from wildfire smoke across primary (<1 day old) and aged (>3 days old) smoke at different concentrations (17). Among the 32 HAPs identified, formaldehyde, benzene, and acrolein – all carcinogenic – were found in primary smoke at levels exceeding what is considered safe for exposure by the Environmental Protection Agency (EPA) (17). While also present in aged smoke, they did not exceed levels considered safe for acute exposure; however, this does not lessen the concern of aged smoke as exposure to even small amounts of carcinogenic pollutants increases the risk for cancer development (17). However, as different chemical compositions exist between primary and aged smoke, it is likely that each result in different underlying biological consequences. These consequences likely include molecular and homeostatic pathway disruptions via mutagenic changes, types of cell apoptosis, or other effector mechanisms. These mechanisms may then trigger a cascade of tissue remodeling, leading to the development of various cancer types, fibrosis and other adverse health outcomes, or allow the tissue to heal itself, depending on the severity of the physiological effects and any comorbidities.

### 3.3 Particulate matter and traveled OA from other hazardous environmental exposures

PMs are studied extensively as the main pollutant making up wildfire smoke. As previously mentioned, many PMs from smoke are toxic based off of their chemical structure and composition; however, particulates themselves cause adverse health effects that are unrelated to their chemical make-up but rather to their size. For example, inhaled ultrafine particulates have increased toxicity compared to their PM_2.5_ counterpart (35). PM_0.1_, a major source of particulate matter from wildfires, can absorb harmful toxicants from smoke and when inhaled, may embed into the lungs for extended timeframes. Furthermore, their smaller size allows for higher particulate infiltration into the bloodstream and increases the possibility of transporting systemically throughout the body and impacting other organs, likely inducing a systemic inflammatory response (35). Indeed, in a 2022 report generated by Heaney et al., unscheduled hospital admissions following wildfire smoke exposure saw an increase in cardiovascular, heart failure, and ischemic heart disease related admissions, and a higher risk for asthma development in children aged 0–5 following exposure (61). Studies such as these demonstrate the dangers of inhaled PM, further highlighting respirable particulates as hazardous regardless of their chemical make-up.

Particulates from burnt materials undergo incomplete combustion, generating VOCs through thermal degradation (i.e., pyrolysis) and off-gassing (62, 63). The released VOCs not only implicate exposed individuals, but also enhance the fire’s volatility through a feedback loop, leading to longer-lasting fires that spread easily over their environment (39). As more acreage is burnt, communities in close proximity are directly exposed to these VOCs. However, previous research has shown that proximity to various environmental exposures is not necessarily more dangerous than being further away from the source as windblown PMs and VOCs travel hundreds to thousands of miles in smoke clouds, remaining in the atmosphere at varying concentrations for unknown periods of time (17). Additionally, as the smoke disperses further away from the source, surrounding communities are likely chronically exposed to a lower dose than if they were nearer to the source – the biological consequences of which are not clearly understood. Furthermore, while health comparisons between POA and SOA from wildfire smoke exposure are not well understood, literature on other hazardous traveled pollutants may provide insight of the biological consequences of such exposures.

In a human based study conducted by Schisler et al. (42), diesel emissions from fuel combustion in vehicles impacted biological pathways related to endothelial cell structural maintenance in coronary arteries. The findings included disruptions to FOXO (Forkhead box, class O) transcription factors – which are crucial for maintaining homeostatic and metabolic cellular processes (64) – providing further explanation of how combustion-derived pollutants impact pulmonary and extrapulmonary toxicity. Furthermore, research on *in vitro* murine alveolar macrophages conducted by Liu et al. revealed that SOA, which make up a large fraction of the VOCs and PM_2.5_ found in fire smoke, lead to oxidative stress (43). Upon exposure to SOA, they observed increased activity of caspase 3/7, a cysteine and apoptotic protease (44). Concomitantly, other reactive oxygen species (ROS) production, such as matrix metalloproteinases (MMPs), was decreased, indicating DNA damage and cell death via apoptosis from SOA exposure (43). Furthermore, changes in expression of MMPs may contribute to changes in lung vasculature as MMPs are a well-known and well documented biomarker of lung angiogenesis (65), another understudied topic in wildfire smoke exposure. These studies allow researchers to understand how innate versus adaptive immunity plays a role in the face of harmful environmental exposures and clearly highlights potential effectors of pathophysiological developments. Identifying these effectors may further indicate that downwind particulates and SOA, or aged smoke, remain toxic as it oxidizes and travels. This sheds light on what physiological consequences may be expected from wildfire smoke exposures. However, whether these effectors in wildfire smoke exposure leads to other biological consequences, such as irreversible tissue damage, cancer development, or other chronic disease development remains unanswered.

Previous air sampling studies from wildfire exposure demonstrated that formaldehyde, a common industrial and environmental pollutant, is significantly increased in the face of wildfires at toxic levels (17). Formaldehyde (FA) is known to trigger lung inflammation, leading to downstream inflammatory disorders and airway remodeling, such as asthma and pulmonary fibrosis (66). FA exposure also plays a role in cardiovascular disease development, where *in vivo* studies have shown that even low amounts of FA exposure can cause endothelial damage and cardiomyocyte apoptosis (67). According to the EPA, FA is the leading carcinogen from over 180 HAPs, and in the atmosphere, is oxidized from VOCs released by fires or biogenic and anthropogenic sources (66, 68). Because SOAs are oxidized from POAs, this FA example further demonstrates a correlation between respiratory disorders and aged smoke exposure, and repeated and chronic exposure to chemicals such as FA greatly increase the risk for further airway remodeling and cancer development (66).

Fortunately, these types of air sampling experiments may allow researchers to recreate smoke exposures in a controlled laboratory environment where the chemical composition of the smoke can be analyzed and compared to what is known from environmental smoke sampling in literature. These techniques may provide the framework needed to begin drawing comparisons between toxic composition of smoke and derived health effects. It is understood that aged smoke has a different chemical makeup than primary smoke, so this may shed light on the adverse health outcomes reported by individuals living in populations further away from the source of the fire, but are still exposed to the aged smoke downwind, and how their health changes from repeated or chronic exposures.

### 3.4 Exposure considerations for occupational workers and surrounding communities

In a study on mental health done by Jung et al. (27), the 2020 wildfire season in California saw increased reports of anxiety and depressive or psychotic episodes. However, similar research regarding respiratory, cardiovascular, and other neurological outcomes are very limited. Current literature, specifically on adverse respiratory health, focuses mainly on lung function in firefighters (4, 5, 13, 14, 25, 34). While findings of decreased lung function from direct wildfire smoke exposure is highly informative (34), it does not provide the needed correlations to the biological mechanisms triggered from smoke exposure in either firefighters or exposed civilians. Furthermore, studies from environmental and occupational PM, diesel emission, and other wildfire hazardous air pollutant exposure suggest that these toxic components may result in adverse cardiovascular, pulmonary, and extrapulmonary health outcomes (17, 42, 43, 48, 49). These studies demonstrate that it is important to conduct research in the same capacity for all environmental exposure types. If similar research existed regarding WLF and WUI exposures, then exposed communities and occupational workers – such as firefighters, farmworkers, and other outdoor workers – may be informed on exposure mitigation and priority treatment could be provided. Unfortunately, at this time, exposed occupational workers continue to be heavily burdened with smoke and other environmental pollution, further increasing their risk for major disease development (48).

Considering the effects of toxic pollutant and particulate exposure, and comparing the known exacerbations of respiratory, neurological, and cardiovascular disease derived from wildfire smoke, we begin to see a more complex picture of the potential underlying health effects from smoke emissions. Literature reviews, clinical cases, and hospital reports show that adverse health outcomes occur from smoke exposure; however, the affected pathways from underlying biological mechanisms are poorly understood and rarely discussed within the literature. Unfortunately, as wildfires can last for weeks to months at a time, occupational firefighters, outdoor farmworkers and civilian populations are chronically exposed to both downwind smoke and smoke generated at the source (48). Because it is well understood what the chemical makeup may entail within these fires as well as what types of diseases some of these toxic chemicals may cause, we can consider that chronic exposure to wildfire toxins from both primary and aged smoke are resulting in (a) over- or under- expression of physiological biomarkers, such as inflammatory cytokine production or gene expression, then consequently leading to (b) chronic pathophysiological changes in tissues – which, to our knowledge, are not addressed in literature – such as damage to the airway epithelium and vasculature bed within the lungs. In other words, the induced health problems from fire exposures are likely a direct consequence of a downstream cascade of reactions within the body that may include biologically disruptive processes – such as DNA damage, cell damage, and even cell death – the pathways of which are currently unknown, especially with regard to long term progressive effects on lung tissue and impacts on lung capacity and function over time. This further addresses the need to understand these processes so the resulting STERDD may be treated or prevented.

## 4 Conclusion

### 4.1 The need for a working experimental system on WUI and aged smoke health comparisons

In 2019, the American Thoracic Society convened to discuss public health implications from wildland fires. It was determined that acute exposure to these emissions causes short-term adverse respiratory effects, especially in those with comorbidities such as asthma and COPD. However, they state that due to the complexity of WUI and WLF exposure types, the long-term impacts of repeated exposures, especially in those chronically exposed—such as firefighters—need further research to draw definitive conclusions and epidemiological correlations (15). The consequences of exposure to chemicals within the smoke are what researchers must consider focusing on in order to understand and explain what changes are occurring at the cellular level within the body from chronic aged and primary smoke exposure. In terms of cardiopulmonary health, the question remains of how these derived respiratory and cardiovascular developments can be treated. How can damage to these structures be prevented, if we do not yet understand the type of physiological damage occurring from WUI emissions? Answering these questions is an immediate goal; however, relying solely on epidemiological studies will not provide a complete picture on which underlying biological and physiological processes are affected from these smoke exposures. We argue that recreating or simulating these fires and studying the phenotypic mechanisms of the physiological and histopathologic changes from these exposures may provide the framework for optimal treatment and prevention.

To date, there are hundreds of hospitalization and epidemiological reports on adverse effects and increased health risks following exposure to wildfires. However, the current literature fails to comprehensively distinguish between burn types, WLF vs. WUI components, fuel types, smoke emissions components, and aged smoke and drawing correlations to specific health outcomes. However, it does provide the necessary information needed to create a novel experimental system. With such a system, researchers can perform specific burn types and expose pre-clinical models to different smoke emissions in an already tested environmental chamber exposure system (ECES) (69, 70). The benefit of such a system is that it allows researchers to conduct continuous whole-body exposures at low doses for extended periods of time, allowing for any physiological changes to take root so histological examination can reveal what these changes may be. Furthermore, by sampling or generating wildfire smoke specific to WLF or WUI fires and analyzing the resulting chemical components, comparisons can be made between lab-generated smoke and environmental air sampling reports. Captured smoke from a lab-controlled technique may allow for exposure to toxic components of the smoke within an experimental system, such as the ECES, at specific concentrations. With this controlled system, conclusions can be drawn to determine specific adverse health outcomes and the underlying biological mechanisms of chronic smoke-derived disease development throughout the lungs, brain, and related vasculature.

## Glossary

CCL2: chemokine ligand 2
COPD: chronic obstructive pulmonary disease
DNA: deoxyribonucleic acid
ECES: environmental chamber exposure system
EPA: Environmental Protection Agency
FA: Formaldehyde
FOXO: Forkhead box, class O
HAPs: hazardous air pollutants
HONO: nitrous acid
ICAM-1: intercellular adhesion molecule-1
IL-1β: interleukin-1beta
IL-5,6,10: interleukin 5,6,10
ILD: interstitial lung disease
MMPs: matrix metalloproteinase
NO_2_: nitrogen dioxide
OA: organic aerosol
PAH: polycyclic aromatic hydrocarbons
PFAS: perfluoroalkyl and polyfluoroalkyl substances
PM: particulate matter
POA: primary organic aerosol
ROS: reactive oxygen species
SOA: secondary organic aerosol
STERDD: smoke-toxin-exposure-related-disease-development
TNF-*α*: tumor necrosis factor-alpha
U.S.: United States
VCAM-1: vascular cell adhesion molecule-1
VOC: volatile organic carbons
WLF: wildland fires
WUI: wildland-urban interfaces
